# Supplementary material for: Novel Driver Strength Index highlights important cancer genes in TCGA PanCanAtlas patients
Source: PeerJ. 2022 Aug 11;10:e13860. doi: 10.7717/peerj.13860 (PMC9375969; doi:10.7717/peerj.13860)
Supplement: Supplemental Information 2 [file peerj-10-13860-s002.zip › COHORTS/genes plots/detailed/2021_8_16_14_9_distribution_events_detailed_females_12.pdf]

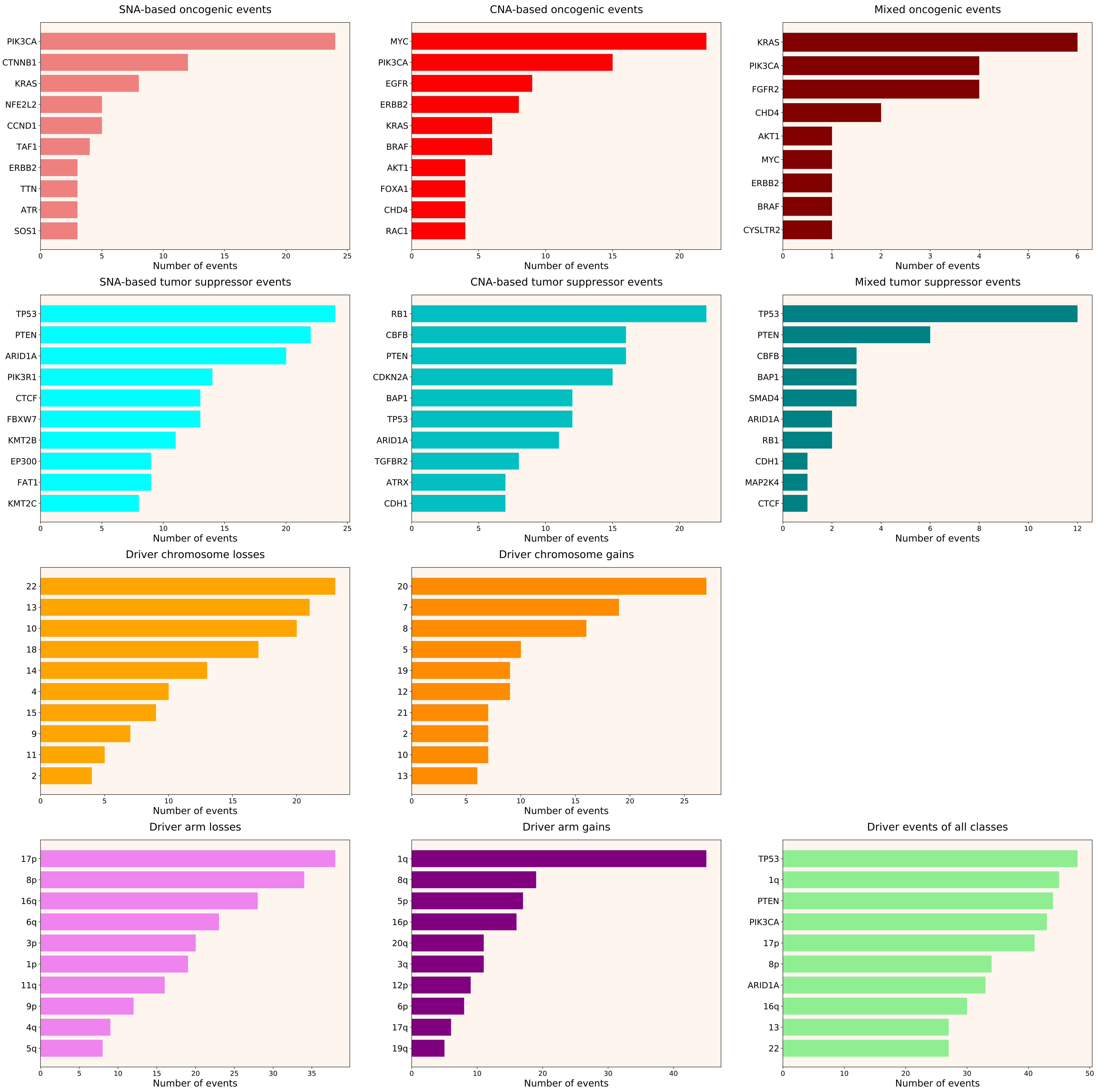

SNA-based oncogenic events
PIK3CA
CTNNB1
KRAS
NFE2L2
CCND1
TAF1
ERBB2
TTN
ATR
SOS1
0 5 10 15 20 25
Number of events
CNA-based oncogenic events
MYC
PIK3CA
EGFR
ERBB2
KRAS
BRAF
AKT1
FOXA1
CHD4
RAC1
0 5 10 15 20
Number of events
Mixed oncogenic events
KRAS
PIK3CA
FGFR2
CHD4
AKT1
MYC
ERBB2
BRAF
CYSLTR2
0 1 2 3 4 5 6
Number of events
SNA-based tumor suppressor events
TP53
PTEN
ARID1A
PIK3R1
CTCF
FBXW7
KMT2B
EP300
FAT1
KMT2C
0 5 10 15 20 25
Number of events
CNA-based tumor suppressor events
RB1
CBFB
PTEN
CDKN2A
BAP1
TP53
ARID1A
TGFBR2
ATRX
CDH1
0 5 10 15 20
Number of events
Mixed tumor suppressor events
TP53
PTEN
CBFB
BAP1
SMAD4
ARID1A
RB1
CDH1
MAP2K4
CTCF
0 2 4 6 8 10 12
Number of events
Driver chromosome losses
22
13
10
18
14
4
15
9
11
2
0 5 10 15 20
Number of events
Driver chromosome gains
20
7
8
5
19
12
21
2
10
13
0 5 10 15 20 25
Number of events
Driver arm losses
17p
8p
16q
6q
3p
1p
11q
9p
4q
5q
0 5 10 15 20 25 30 35
Number of events
Driver arm gains
1q
8q
5p
16p
20q
3q
12p
6p
17q
19q
0 10 20 30 40
Number of events
Driver events of all classes
TP53
1q
PTEN
PIK3CA
17p
8p
ARID1A
16q
13
22
0 10 20 30 40 50
Number of events
